# Recording and Reporting of Antimicrobial Resistance (AMR) Priority Variables and Its Implication on Expanding Surveillance Sites in Nepal: A CAPTURA Experience

**DOI:** 10.1093/cid/ciad581

**Published:** 2023-12-20

**Authors:** Sanju Maharjan, Patrick Gallagher, Manish Gautam, Hea Sun Joh, Mohammad Julhas Sujan, Ahmed Taha Aboushady, Soo Young Kwon, Sanjay Gautam, Madan Kumar Upadhyaya, Runa Jha, Jyoti Acharya, William R MacWright, Florian Marks, John Stelling, Nimesh Poudyal

**Affiliations:** Public Health Research, Anweshan Private Limited, Lalitpur, Nepal; Public Health Surveillance Group, LLC, Princeton, New Jersey, USA; Public Health Research, Anweshan Private Limited, Lalitpur, Nepal; International Vaccine Institute, Seoul, Republic of Korea; International Vaccine Institute, Seoul, Republic of Korea; International Vaccine Institute, Seoul, Republic of Korea; Brigham & Women's Hospital, Harvard Medical School, Boston, Massachusetts, USA; International Vaccine Institute, Seoul, Republic of Korea; International Vaccine Institute, Seoul, Republic of Korea; Research & Collaboration, Anka Analytica, Melbourne, Australia; Ministry of Health and Population, Government of Nepal, Kathmandu, Nepal; National Public Health Laboratory, Department of Health Services, Ministry of Health and Population, Kathmandu, Nepal; National Public Health Laboratory, Department of Health Services, Ministry of Health and Population, Kathmandu, Nepal; Public Health Surveillance Group, LLC, Princeton, New Jersey, USA; International Vaccine Institute, Seoul, Republic of Korea; Cambridge Institute of Therapeutic Immunology and Infectious Disease, University of Cambridge School of Clinical Medicine, Cambridge, United Kingdom; Heidelberg Institute of Global Health, University of Heidelberg, Heidelberg, Germany; Madagascar Institute for Vaccine Research, University of Antananarivo, Madagascar; Brigham & Women's Hospital, Harvard Medical School, Boston, Massachusetts, USA; International Vaccine Institute, Seoul, Republic of Korea

**Keywords:** antimicrobial resistance, Nepal, NPHL, network

## Abstract

Data on antimicrobial resistance (AMR) from sites not participating in the National AMR surveillance network, conducted by National Public Health Laboratory (NPHL), remain largely unknown in Nepal. The “Capturing Data on Antimicrobial Resistance Patterns and Trends in Use in Regions of Asia” (CAPTURA) assessed AMR data from previously untapped data sources in Nepal. A retrospective cross-sectional data review was carried out for the AMR data recorded between January 2017 and December 2019 to analyze AMR data from 26 hospital-based laboratories and 2 diagnostic laboratories in Nepal. Of the 56 health facilities initially contacted to participate in this project activity, 50.0% (28/56) signed a data-sharing agreement with CAPTURA. Eleven of the 28 hospitals were AMR surveillance sites, whereas the other 17, although not part of the National AMR surveillance network, recorded AMR-related data. Data for 663 602 isolates obtained from 580 038 patients were analyzed. A complete record of the 11 CAPTURA priority variables was obtained from 45.5% (5/11) of government hospitals, 63.6% (7/11) of private hospitals, and 54.6% (6/11) of public-private hospitals networked with NPHL for AMR surveillance. Similarly, 80% (8/10) of clinics and 54.6% (6/11) of laboratories outside the NPHL network recorded complete data for the 10 Global Antimicrobial Resistance and Use Surveillance System (GLASS) priority variables and 11/14 CAPTURA priority variables. Retrospective review of the data identified areas requiring additional resources and interventions to improve the quality of data on AMR in Nepal. Furthermore, we observed no difference in the priority variables reported by sites within or outside the NPHL network, thus suggesting that policies could be made to expand the surveillance system to include these sites without substantially affecting the government's budget.

## Teaser

We reviewed antimicrobial resistance (AMR) data from multiple sites within and outside the national surveillance program to strengthen AMR surveillance.

WHONET/BacLink supports AMR data management and could be used to expand surveillance sites for AMR and allocate resources strategically.

The National Public Health Laboratory (NPHL) conducts laboratory-based antimicrobial resistance (AMR) surveillance in Nepal. It also serves as the National Coordination Centre and National Reference Laboratory for the same purpose. In 1999, the NPHL initiated AMR surveillance at nine sites. Currently, 26 microbiology laboratories routinely report their data on bacterial culture and AMR findings through this network [1]. The selection criteria for the sentinel sites are not well defined, and participation remains entirely voluntary. However, the NPHL monitors laboratories with bacterial culturing facilities and encourages them to generate higher volumes of data, although the exact expected volume remains undefined. The AMR reporting system is gradually being streamlined under the NPHL's leadership, but issues continue to be encountered regarding receiving complete, consistent, and uniformly stored data from the surveillance sites [2], owing to a shortage of dedicated and trained data handling personnel, frequent staff turnover, limited access to quality reagents and storage facilities, power outages, insufficient funding due to competing priorities, a lack of adequate training opportunities and infrastructure, and frequent policy changes [3, 4].

The NPHL coordinates with national and international agencies, including the Fleming Fund, to conduct AMR surveillance activities. “Capturing Data on Antimicrobial Resistance Patterns and Trends in Use in Regions of Asia” (CAPTURA) is a project funded by the UKaid, with a specific focus on historical data collection on AMR and AMU in South and Southeast Asia, among other initiatives addressing the global threat of AMR [5]. CAPTURA has been implemented in 6 countries in South Asia (Nepal, India, Bangladesh, Sri Lanka, Bhutan, and Pakistan) and 6 countries in Southeast Asia (Indonesia, Timor-Leste, Papua New Guinea, Laos, Vietnam, and Myanmar).

The primary aims of CAPTURA are to increase the volume of available data on AMR and the use of antimicrobial agents; assess the quality of the data; report meta-data to provide regional and interregional context; analyze the data; and ensure that the findings are disseminated locally, regionally, and globally [5]. Herein we present local experience in collecting and managing AMR-related data from sites within or outside the NPHL network during the implementation of the CAPTURA project.

## METHODS

### Study Design and Settings

**Figure 1.**
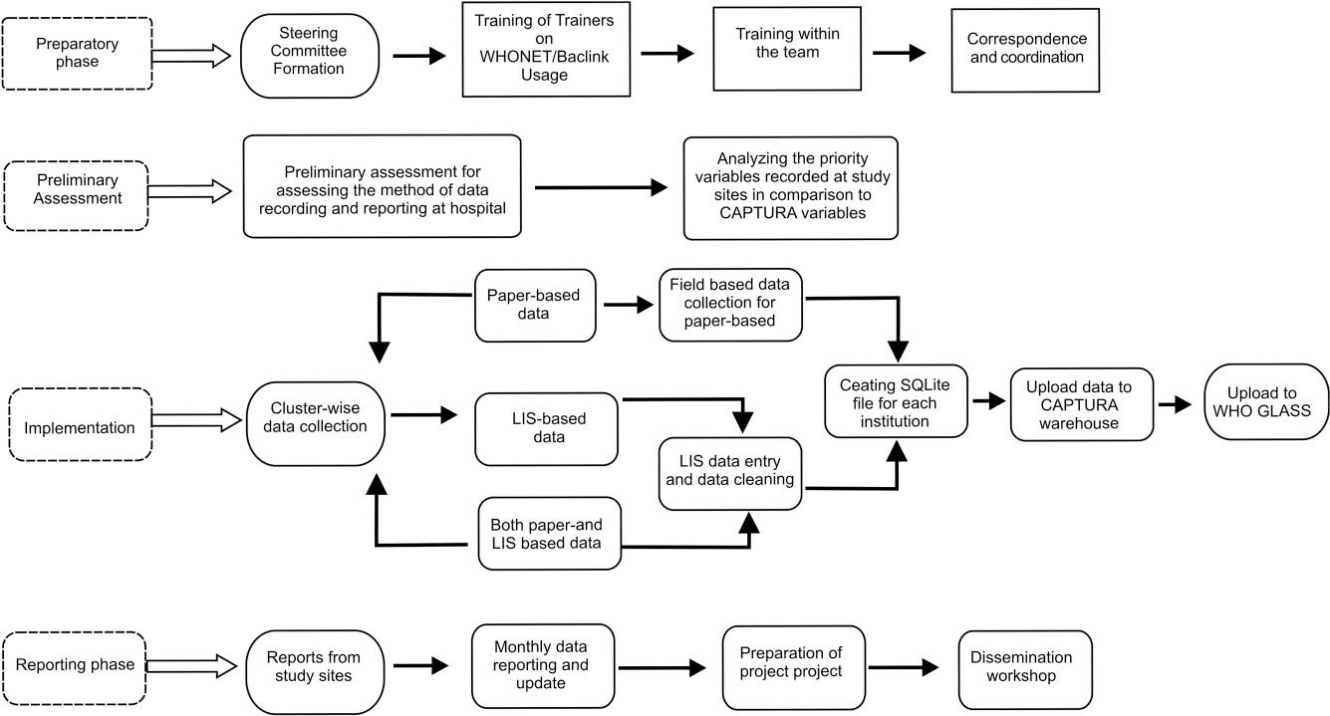
The CAPTURA study operational framework in Nepal. Abbreviations: CAPTURA, Capturing Data on Antimicrobial Resistance Patterns and Trends in Use in Regions of Asia; GLASS, Global Antimicrobial Resistance and Use Surveillance System; WHO, World Health Organization.

The CAPTURA project consortium in Nepal was led by the International Vaccine Institute (IVI) with Public Health Surveillance Group (PHSG) as a co-partner, the Nepal-based public health research firm Anweshan, and stakeholders including the Ministry of Health and Population (MoHP). A retrospective cross-sectional data review was conducted to analyze the AMR data collected between January 2017 and December 2019 from 26 hospital laboratories and 2 diagnostics laboratories in 10 districts in 5 provinces. For inclusion of the sites, MoHP provided a list of 56 laboratories with microbial culture facilities. Each site was contacted and approached for agreement to share AMR data with the project team and was included in the data collection. The obtained data were analyzed for microbiological and clinical information associated with AMR. A detailed list of the data recording system (Table 1) and a schematic of the operational framework showing three different phases and project activities (Figure 1) are provided.

**Table 1.** Data Clusters and Data Collection Methods at Various Study Sites

Data Type of Hospital	Type of Study Sites (NPHL Network)						Total
	AMR Surveillance Network			Outside Network			
	Government Hospital^a^	Private Hospital^b^	Public-private^c^	Government Hospital	Private Hospital	Clinic/laboratory	
LIS^d^	3	1	3	0	3	0	10
Paper-based	2	0	0	1	2	1	6
Both	0	1	0	4	5	1	11
WHONET	0	1	0	0	0	0	1
**Total**	**5**	**3**	**3**	**5**	**10**	**2**	**28**

Abbreviations: LIS, laboratory information system; NPHL, National Public Health Laboratory.

^a^Hospitals under federal or state government control, fully funded by the government and operating solely on taxpayer money.

^b^Hospitals financed and operated by a person or groups of owners.

^c^Hospitals partially funded by the government.

^d^LIS system (Dolphin, MIDAS, HOPE, Medipro, VITEK, HIS, or Healthy life).

### Data Collection

**Figure 2.**
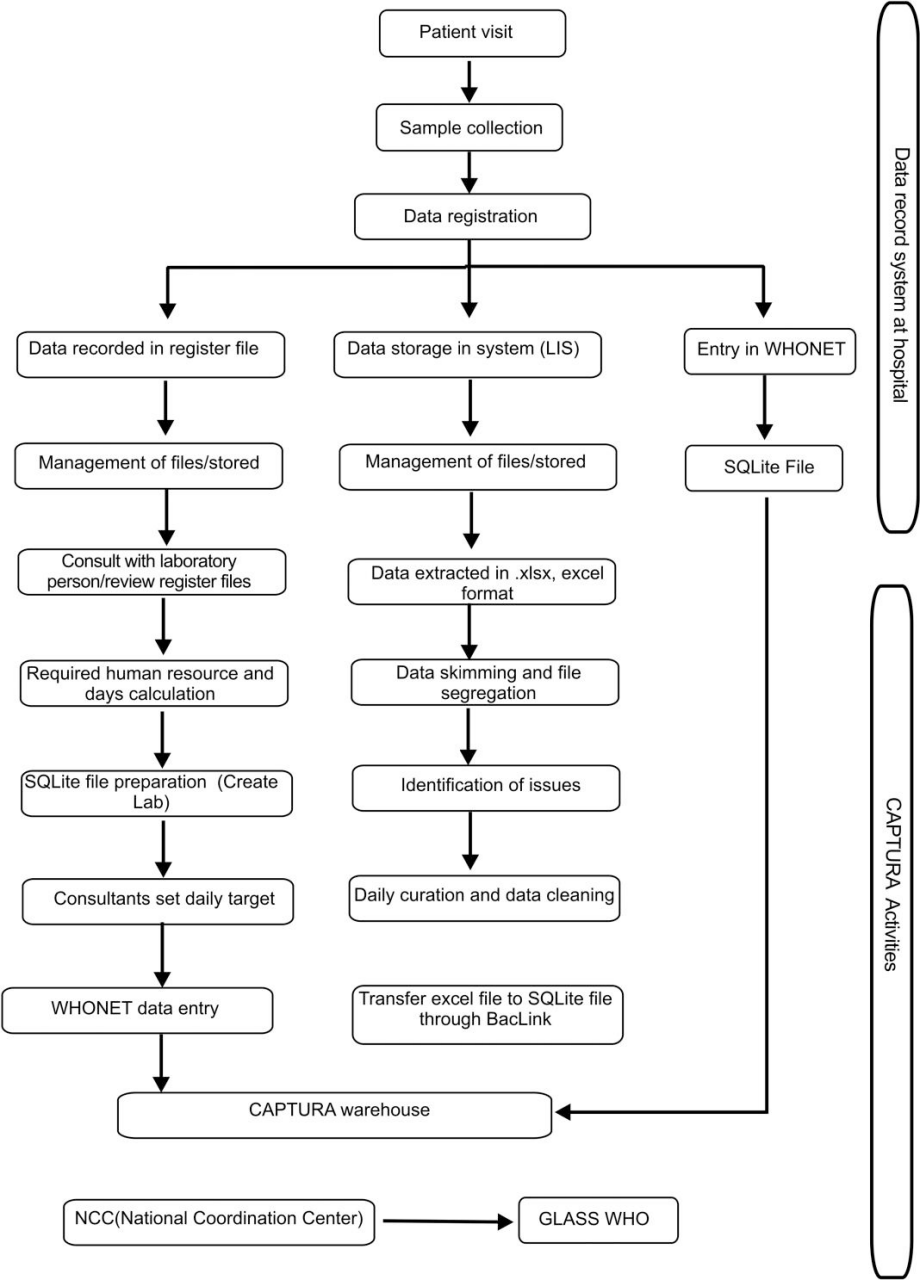
Data collection process. Abbreviations: CAPTURA, Capturing Data on Antimicrobial Resistance Patterns and Trends in Use in Regions of Asia; LIS, laboratory information system; WHO, World Health Organization.

A total of 56 sites were recommended by the MoHP and were chosen based on their potential to further scale AMR capacity building activities. All of these sites were officially approached for participation in the CAPTURA project, of which only 50% (28/56) agreed to participate. A data transfer agreement was signed with each facility to participate in the process, and data were collected soon thereafter. All hospitals, clinics, and laboratories selected as CAPTURA project sites were categorized into four clusters based on their method of data management: paper-based (cluster I); laboratory information system (LIS) (cluster II); both paper-based and LIS (cluster III); and WHONET system (cluster IV) (https://whonet.org/). Before initiation of the data collection process, a preliminary list of variables (antibiotic codes, and microbiological and clinical information) was prepared in consultation with microbiologists. A dedicated team of 4 members extracted information from paper-based entries, whereas 2 team members collected data from the LIS. The data collection process is illustrated in Figure 2. The data collected through paper-based records were directly uploaded into the WHONET system through creation of a laboratory in WHONET software. Uniformity in variable columns and formats for recording data was maintained within the shared files from across the hospitals throughout the process. No personal identifiers were recorded, and the confidentiality of the data stored in the cloud and the system was ensured. The data were recorded in Microsoft Excel (.xlsx) format and R software (https://www.r-project.org/about.html). For compatibility, the Microsoft Excel files were curated and converted to SQLITE format with BacLink and uploaded to the WHONET system.

### AMR Data Quality

To ensure better data governance, the quality of the retrieved AMR data was maintained on the basis of the following factors.

### Consistency

To ensure consistency in the data uploaded into the system through BacLink, this study adopted the variables and their datatype formats, according to CAPTURA software requirements (Supplementary Data1). Specimen and sample details were cleaned and made consistent throughout the shared files, and the recorded organisms were split into rows based on the number of organisms extracted. Likewise, the AST reports generated for each antibiotic were formatted to include a single column for each antibiotic type in the record. Additionally, granularity within the recorded variables was maintained by rigorous cleaning of the data for each file extracted from the individual hospitals.

### Validity

Data were validated in multiple stages. First, data from each hospital were extracted, skimmed, and curated according to CAPTURA's required variables and formats before being uploaded to the WHONET system. Next, WHONET software was used to search for discrepancies, and data were rejected or flagged as erroneous when the following occurred:

- One or more variables were missing.
- The order of variables differed from the WHONET format.
- The files had missing values.
- The values in the files were not included in the coded values list.
- Variable abbreviations differed within the submitted file.

Hospitals and laboratories with identified erroneous data and missing values were followed up for maintaining data accuracy and consistency during the data cleaning process once rejected through the WHONET system.

### Completeness

Data completeness was defined according to the data recorded for CAPTURA priority variables (Supplementary Data1). A total of 17 variables were recorded, 11 of which were recorded as CAPTURA priority variables and the remaining six of which were recorded as other variables. However, the information recorded was incomplete and inconsistent for most of the variables.

### Integrity

Data integrity was ensured by provision of rigorous WHONET training to consultants; thorough review and checking of the data uploaded to the software by the project microbiologist; and consultation with laboratory personnel and hospital authorities regarding missing, incomplete, or incorrect data.

### Granularity

The LIS files extracted from each hospital were cleaned and managed separately, but after being uploaded to WHONET, they were merged or stored as a single large file. Although the hospitals used similar data recording variables, the files’ granularity was not adequately maintained. Only a few hospitals used the same format to record data, whereas the data structures varied across other hospitals, because they were customized according to the facility's needs. This study strictly ensured the granularity of the data as part of the cleaning and validating process, and a consistent format was used to upload the files to WHONET.

## RESULTS

### Facilities Covered

Of the total facilities contacted, 50% (28/56) agreed to share their data with the project team. Most of the enrolled hospitals were privately owned, and for better representation of AMR data, the preliminary list of 56 hospitals included institutions from all 7 provinces. However, after purposive selection of the hospitals and laboratories based on CDA/DTA signed for participation, the study sites were disproportionately distributed geographically. For instance, sites from Madesh and Karnali provinces declined to participate for multiple reasons (described in detail in the Limitations section), thus leading to the exclusion of hospitals and laboratories from those 2 regions. In total, 28 institutions were selected from 5 provinces.

Among the 28 selected hospitals, 11 were AMR surveillance sentinel sites reporting to the NPHL which included 5 governmental hospitals, 3 private hospitals, and 3 public/private institutions. The additional 17 hospitals were not included in the AMR surveillance network but had been recording AMR-related data. Table 2 shows the details of the facilities covered.

**Table 2.** Types of Hospitals Within or Outside the AMR Surveillance Network Participating in the Study

	Type of Study Sites						
	AMR Surveillance Network			Outside Network			
Province	Government Hospital^a^	Private Hospital^b^	Public-private^c^	Government Hospital	Private Hospital	Clinic/laboratory	Total
Province 1	1	0	0	0	2	0	**3**
Bagmati province	2	1	2	4	7	2	**18**
Gandaki province	0	1	0	0	0	0	**1**
Lumbini province	2	1	0	0	1	0	**4**
Sudurpaschim province	0	0	1	1	0	0	**2**
**Total**	**5**	**3**	**3**	**5**	**10**	**2**	**28**

Abbreviation: AMR, antimicrobial resistance.

^a^Hospitals under federal or state government control, fully funded by the government and operating solely on taxpayer money.

^b^Hospitals financed and operated by a person or groups of owners.

^c^Hospitals partially funded by the government.

### Data Volume and Quantity

Data from 663 602 isolates obtained from 580 038 patients, mostly originating from private hospitals (n = 357 166), were uploaded to the CAPTURA warehouse. Here the total number of isolates refers to the number of samples collected from patients for bacteriological investigation. A single patient might have multiple samples collected and tested, or, because a single sample can contain various pathogens, this number might differ from the total number of patients. In general, the total number of patients is a valuable metric for tracking disease spread, and the total number of isolates indicates a specific pathogen type causing disease. Regarding specimen type, the isolates originated from blood, urine, body fluids, sputum, stool, swabs, catheters, and other devices, where the highest numbers were recorded for urine, followed by blood and sputum. The highest numbers of registered patients and isolates were in the public-private sentinel sites, where patients also had multiple isolates. Second highest number were from government hospitals that were not part of the NPHL network, where patients with no multiple isolates were encountered (Supplementary Data2).

In this study, private hospitals tested more antibiotics (maximum and minimum of 61 and 31 antibiotics tested, respectively) as compared to public-private hospitals (maximum and minimum of 45 and 17 antibiotics tested, respectively). Likewise, among the 11 sentinel sites, a single hospital was reported to have tested a maximum of 61 antibiotics and a minimum of 17 antibiotics. For the remaining 17 hospitals, the number of antibiotics tested at a single laboratory ranged from 32 to 58.

#### Priority Variables

A total of 48 variables, with 8 optional variables, were included as CAPTURA variables (Supplementary Data2). Eleven priority variables, including age, sex, microorganism isolated, specimen type, and antibiotic test results, were recorded at all hospitals (Table 3). The least recorded variables were department and location (units within the department). Some CAPTURA priority variables, such as specimen date and location, were not recorded at both sentinel and non-sentinel sites. Antibiotic susceptibility test (AST) reports were generated at all hospitals, along with specimen type and organism details.

**Table 3.** Variables of Interest Recorded During the Study

CAPTURA Priority Variable	Type of Hospital					
	AMR Surveillance Network			Outside Network		
	Government Hospital	Private Hospital	Public-private	Government Hospital	Private Hospital	Clinic/laboratory
	n = 5	n = 3	n = 3	n = 5	n = 10	n = 2
Age	5	3	3	5	10	2
Sex	5	3	3	5	10	2
Location	2	1	1	1	2	1
Healthcare facility admission date (inpatient)	0	0	0	0	0	0
Healthcare facility visit date (outpatient)	0	0	0	0	0	0
Specimen date	4	1	1	3	7	2
Specimen type	5	3	3	5	10	2
Organism	5	3	3	5	10	2
Antibiotic result	5	3	3	5	10	2
Other variables recorded						
Patient identification number	3	2	2	1	5	0
Specimen number	2	3	3	4	7	1
Location type	3	2	2	1	5	1
Department	0	3	1	1	3	0
Country name	5	3	3	5	10	2
Institution Name	5	3	3	5	10	2

Abbreviations: AMR, antimicrobial resistance; CAPTURA, Capturing Data on Antimicrobial Resistance Patterns and Trends in Use in Regions of Asia.

Ten GLASS priority were included (Table 4). However, no information was available to identify classes of antibiotic resistance such as detection of methicillin-resistant *Staphylococcus aureus* in any hospital/laboratories. Likewise, patient identification numbers were unavailable in the laboratory/clinic records.

**Table 4.** GLASS Priority Variables

GLASS Priority Variable	Type of Hospital					
	AMR Surveillance Network			Outside Network		
	Government Hospital	Private Hospital	Public-private	Government Hospital	Private Hospital	Clinic/laboratory
	n = 5	n = 3	n = 3	n = 5	n = 10	n = 2
Country name	5	3	3	5	10	2
Sample origin	5	3	3	5	10	2
Patient identification number	3	2	2	1	5	0
Age	5	3	3	5	10	2
Sex	5	3	3	5	10	2
Specimen date	4	1	1	3	7	2
Specimen type	5	3	3	5	10	2
Organism	5	3	3	5	10	2
Antibiotic result	5	3	3	5	10	2
Antibacterial agent	0	0	0	0	0	0

Abbreviations: AMR, antimicrobial resistance; GLASS, Global Antimicrobial Resistance and Use Surveillance System.

#### Data Dictionary

None of the hospitals-maintained data dictionaries, although LIS systems such as Midas, Healthy Life, Danphe, Hangout, Sukra, and Dolphin were used at the institutions. Similarly, hospitals that continue to record data on paper lacked data dictionaries. Therefore, this study used CAPTURA's data variable format to maintain consistency.

#### Data Consistency and Completeness

The AMR data collected from the hospitals were inconsistent and incomplete. For example, none of the hospitals used similar data recording format, and the priority variables differed across institutions. Consequently, the data extracted from their LIS were neither well-organized nor systematic. Furthermore, the data recording system in the hospital servers prevented us from obtaining the year specific information for each specimen and sample type. Additionally, because the priority variables for recording AMR data varied across the hospitals, the recorded data varied likewise.

Comparing hospital categories, the completeness of data for CAPTURA variables was relatively higher (63.6%) in private hospitals within the NPHL network. In contrast, the completeness of data for GLASS variables were relatively higher (80%) in clinics/laboratories outside the network (Supplementary Data3). Similarly, 45.5% of government hospitals, 63.6% of private hospitals, and 54.6% of public-private hospitals under the NPHL network reported complete data for 11 priority variables. Likewise, 70.0% of all health facilities within the network had practised recording all ten GLASS priority variables. However, 80.0% and 54.6% of clinics and laboratories outside the NPHL network recorded all ten GLASS priority variables and 11 CAPTURA priority variables, respectively (Tables 3 and 4).

## DISCUSSION

The CAPTURA project collected data recorded from January 2017 to December 2019 on AMR from 28 healthcare facilities of Nepal. The data collection period spanned from August 2020 to June 2021. Six facilities relied on paper-based data recording, whereas 10 facilities used a LIS to record and monitor AMR data. The rest of the facilities used both paper and LIS recording method. This parallels the situation observed in low- and middle-income countries where laboratories depended more on paper-based methods for data collection with limited use of LIS system [6, 7]. We identified 11 hospitals using LIS and paper-based data recording methods. However, technical limitations prevented us from accessing the LIS data from 3 of these hospitals, so the records were manually uploaded into WHONET system from the paper records. And it is similar with the barriers identified in previous studies in low- and middle-income countries, including a lack of data standards, a shortage of trained IT personnel, technical problems, and difficulties with system interoperability in electronic data recording [6,8].

The NPHL analyzes data from sentinel sites for submission to GLASS [2]. Our study locations consisted of 11 sentinel sites under the NPHL: 5 government-run, 3 private, and 3 public-private sites. Of these 11 hospitals, 7 recorded data in a LIS, 2 were paper-based, 1 used both LIS and paper-based data methods, and 1 used WHONET. This study indicated that AMR data management varied widely among facilities, some of which had relatively better reporting and recording mechanisms. These variations were due to the different levels of technology used, including the availability of electronic medical records, mainly the LIS. Some facilities had developed superior electronic medical records systems that allowed for better AMR data management, whereas others relied on older or less sophisticated methods, such as paper-based record keeping. Similarly, some facilities had staff dedicated to data management, including that of AMR data, whereas others had limited dedicated resources. The facilities had different levels of acceptance among staff as well as resource availability for AMR data management. Although trainees were excited to learn and adopt the technology, resource limitation and multiple job duties posed severe constraint. Despite the varying approaches to data management approaches, the key variables of primary interest to the GLASS and NPHL remained nearly the same across hospitals (82.0% [9/11]), mainly regarding their reporting to the NPHL. Consequently, this study proposes that the government has the potential to expand sentinel sites without significant resource allocation towards site identification. Instead, by offering training course of 3 or more days and refresher courses to laboratory personnel at each facility, accompanied by practical sessions, the government could establish a broader network of sentinel sites in a sustainable and economically efficient manner. This would be facilitated by having a well-trained cadre of laboratory personnel.

In this study, accessing data from sentinel sites was relatively convenient in terms of time and resources. Sentinel sites provided electronic access to data, whereas data from non-sentinel sites needed to be manually uploaded to the WHONET system, in a more time-consuming and labour-intensive process. Non-sentinel sites also faced challenges such as weak internet connections, a lack of necessary computer hardware and storage units, and a shortage of adequately trained personnel.

Although NPHL annually reports ten GLASS variables, 14 variables were included in our study, based on the CAPTURA project's Country Implementation Plan (CIP). Private hospitals, compared with government hospitals, reported more than twice the number of isolates, possibly because these sites typically serve more patients with more resources and capacity and therefore have more samples to test for isolates. We identified incompleteness and inconsistencies in the data collected through LIS and paper-based methods. For example, information on specimen type, microorganisms reported, and details of AST results were missing, and, more importantly, data were not uniformly recorded. Of the 11 CAPTURA and 10 GLASS priority variables, complete data were obtained in 45%–80% of cases; data consistency could not be measured, because separate files were maintained for different specimens. A previous report from Bagmati Province has indicated 88%–100% data completeness and 77%–92% consistency for data received at the NPHL [2]. The current study also found that the misplacement of registers, and improper file handling and storage were the main reasons for data unavailability/incompleteness.

Furthermore, consistency in available data was also lacking within institutes, mainly because of a lack of data standardization by the government, including the MoHP [9]. Because the LIS records, stores, and manages laboratory data—including test results, patient records, and laboratory management—hence, consistent and complete data input in a LIS is essential for sustainable AMR surveillance [10]. The data can then be used to track the spread of antimicrobial resistance in a population and assess the efficacy of new treatments. In addition, a comprehensive and consistent view of AMR-related data can help identify trends in antibiotic resistance and inform public health decisions. Similarly, the data collected and managed through a LIS can be used to analyse the effects of interventions, such as antibiotic stewardship programs, on trends in antibiotic resistance. Finally, a LIS can be used to monitor the implementation of national and international guidelines regarding the appropriate use of antibiotics. By providing a complete picture of the evolution of AMR, data managed via a LIS can be used to inform and guide policy decisions and help shape future strategies for addressing AMR.

Data recording was inconsistent for almost all variables, and extensive cleaning was required. In addition, the recording system for tracking the use of antibiotics was not available in the hospitals, and patient location and location types were not correctly recorded in most cases. Maintaining data consistency is crucial for AMR studies to enable geographically tailored design and implementation of interventions.

In addition, many hospitals were not correctly recording microorganisms at the species level or not performing necessary resistance marker testing, such as for extended-spectrum beta-lactamase, methicillin-resistant *S. aureus*, methicillin-sensitive *S. aureus*, and vancomycin-resistant enterococci. These markers can provide important information on the resistance patterns of specific organisms and can help guide treatment decisions. Not performing these tests could result in a lack of understanding of the resistance patterns in a particular hospital or region, thus ultimately leading to inadequate treatment and the spread of antibiotic-resistant microorganisms. Eventually, performing resistance marker test can have significant implications for understanding resistance patterns and treatment decisions. The methods used to isolate and identify bacterial isolates were not recorded. Moreover, discordant results were found for quinolone, fluoroquinolones, aminoglycosides, beta-lactam groups, and their inhibitor antibiotics. Proper monitoring is necessary in case of laboratory testing errors. Monitoring data can be used to encourage vigilance in emerging infectious disease outbreaks but have not been applied for this purpose to date.

The current sentinel surveillance sites for AMR in Nepal are insufficient to provide an accurate picture of the prevalence of resistance in the country [7]. Therefore, the number of sentinel sites must be expanded, and a broader range of bacteria must be included to provide a more comprehensive picture of the problem. Additionally, more data must be collected on the use of antibiotics in community and healthcare settings, to help identify possible sources of resistance.

## STRENGTHS AND LIMITATIONS

This study is the first national effort to our knowledge to identify, digitize, and analyse historical AMR data, while giving facilities complete data ownership. This approach allows for a detailed examination of resistance patterns and trends over time, thereby providing essential insights into the evolution of AMR. Furthermore, the active participation of the facilities in considering their findings and using the data for informed decision-making is a key strength ensuring the sustainability of the result generated and its practical application.

This study has several limitations. First, the study was limited because complete data were gathered for 2017–2019, but data for subsequent years were not included. Furthermore, the data sources were chosen purposively on the basis of the health facilities and laboratories identified by the MoHP; therefore, the study's findings cannot be considered representative of the entire country. Although the study encompassed all 5 provinces and included a wide range of institutions, it is important to note that hospitals from 2 provinces, namely, Madesh and Karnali province, were not included. Despite making efforts to engage with major hospitals in these provinces, we were unable to collect data from them as they did not reach to a consensus on signing the Data Transfer Agreement (DTA) for support. However, it is worth mentioning that we were able to gather substantial data from 28 institutions, which provides a valuable snapshot of the country's baseline antimicrobial resistance (AMR) situation.

## CONCLUSIONS AND FUTURE PERSPECTIVES

Based on the results and observations in this study, we conclude that an urgent need exists for a sustainable and resilient AMR surveillance system with a primary focus on building a uniform data recording method. For example, WHONET/BacLink have led to a breakthrough in maintaining the data structure and approach necessary to collect, analyze, comprehend, and disseminate AMR data. These may potentially also be beneficial in planning, designing, and developing programs and policies to control the global surge in AMR.

The government could use the findings of this study to its advantage. The finding of no significant difference in how facilities report AMR data and priority variables indicates that the government could easily expand its surveillance sites without investing in identifying individual facilities. The government could use existing data to assess the prevalence of AMR and other variables across the healthcare system. The data could further be used to inform decisions regarding where to expand surveillance sites or to identify existing sites for potential expansion or modification. Moreover, the government could use the data to identify areas requiring additional resources or interventions to generate quality AMR data.

## Supplementary Data

Supplementary materials are available at *Clinical Infectious Diseases* online. Consisting of data provided by the authors to benefit the reader, the posted materials are not copyedited and are the sole responsibility of the authors, so questions or comments should be addressed to the corresponding author.

## Supplementary Material

ciad581_Supplementary_Data

## Notes

***Disclaimer.*** The views expressed here are those of the authors and not necessarily those of the UK Department of Health and Social Care or its management agent, Mott MacDonald; and, International Vaccine Institute.

***Supplement sponsorship.*** This article appears as part of the supplement “Capturing Data on Antimicrobial Resistance Patterns and Trends in Use in Regions of Asia (CAPTURA),” funded by the Department of Health and Social Care's Fleming Fund using UK aid.

***Financial support.*** This work was supported by the Fleming Fund regional grants (grant numbers FF10-135 and FF11-139). The Fleming Fund is a UK aid investment to support surveillance of antimicrobial resistance in low- and middle-income countries in Africa and Asia. The regional grants program is managed by Mott MacDonald Ltd, the Fleming Fund Grants Management Agent.
