# Supplementary material for: Recording and Reporting of Antimicrobial Resistance (AMR) Priority Variables and Its Implication on Expanding Surveillance Sites in Nepal: A CAPTURA Experience
Source: Clin Infect Dis. 2023 Dec 20;77(Suppl 7):S560–8. doi: 10.1093/cid/ciad581 (PMC10732557; doi:10.1093/cid/ciad581)
Supplement: ciad581_Supplementary_Data [file ciad581_supplementary_data.docx]

**Supplemental Table 1**: CAPTURA priority variable

| CAPTURA AMR DATA VARIABLES | | | | | |
| --- | --- | --- | --- | --- | --- |
| Variable | Sub-Category | Sharing | | | Remarks |
|  |  | Y | N | N/A |  |
| Country |  |  |  |  |  |
| Name of Patient |  |  | No |  | CAPTURA only collects de-identified data |
| Contact details of patient | Address Emails  Phone Number |  | No |  | CAPTURA only collects de-identified data |
| Sample Origin |  |  |  |  | Human |
| Patient Identification  Number |  |  |  |  | Patient ID |
| Date of Birth |  |  |  |  | DD/MM/YYYY |
| Age |  |  |  |  | Specimen Date – Date of Birth |
| Sex | Male  Female |  |  |  |  |
| Institution |  |  |  |  | Name of Facility |
| Level of Healthcare Facility | Primary Secondary  Tertiary |  |  |  |  |
| Geographical Location of  Healthcare facilities | Address  GPS |  |  |  |  |
| Type of Healthcare facility | Public Private NGO  Others |  |  |  |  |
| Healthcare Facility Admission Date (if applicable)/Date of first visit to healthcare  Facility |  |  |  |  |  |
| Department |  |  |  |  |  |
| Patient Location |  |  |  |  |  |
| Patient Location |  |  |  |  |  |
| Admission Diagnosis |  |  |  |  |  |
| Antibiotics received/used.  prior to culture |  |  |  |  |  |
| Clinical Symptom at the time  of Specimen Collection |  |  |  |  |  |
| Specimen Number |  |  |  |  | Specimen ID |
| Specimen type |  |  |  |  |  |
| Specimen Date |  |  |  |  | DD/MM/YYYY |
| Reason for Specimen | Diagnostic |  |  |  |  |
| Collection |  |  |  |  |  |
| Antibiotics Prescribed After  Collection of Specimen |  |  |  |  |  |
| Method Used for Bacterial  Isolation and Identification |  |  |  |  |  |
| Culture result |  |  |  |  |  |
| Organism Isolated |  |  |  |  | Name of microorganism species level |
| Typing Result | Serotype Genotype  Not specified |  |  |  |  |
| Method Used for Antibiotic  Susceptibility |  |  |  |  |  |
| Laboratory Standard  Followed (CLSI, EUCAST etc.), year of guideline |  |  |  |  |  |
| Antimicrobial Susceptibility Test Result | Disk Diffusion  MIC  E-test Others |  |  |  | Antibiotic disk with potency Zone Diameter  Value |
| Beta Lactamase test | Positive  Negative |  |  |  |  |
| ESBL | Positive  Negative |  |  |  |  |
| MRSA | Positive  Negative |  |  |  |  |
| Other |  |  |  |  |  |
| External Quality Assessment  Scheme used by Laboratory |  |  |  |  |  |
| Change to Initial Therapy |  |  |  |  | Antibiotics Prescribed to the Patient After Culture Report |
| Confirmed Diagnosis (after  laboratory investigation) |  |  |  |  |  |
| Date of Discharge (if  applicable) |  |  |  |  |  |
| Patient Outcome | Improved Died Referred  Others |  |  |  |  |
| Date of Death (If applicable) |  |  |  |  | Optional |
| Cause of Death (if applicable) |  |  |  |  | Optional |
| Co-morbidities |  |  |  |  |  |
| Additional/Recurrent admissions | Site Unit Date  Diagnosis  Outcomes |  |  |  | Optional |
| Additional/Recurrent isolates/Infections | Site Unit Date  Diagnosis Outcomes |  |  |  | Optional |
| For Neonates (1 month or  less) | Prematurity  Birthweight |  |  |  | Optional |
| Patient Socio-economic  status |  |  |  |  | Optional |
| Patient level of education |  |  |  |  | Optional |
| Patient ethnicity |  |  |  |  | Optional |

**Supplemental Table 2**: Distribution of patient and bacterial isolate data

| **Type of hospital** | | **Number of isolates** | | **Number of patients** | |
| --- | --- | --- | --- | --- | --- |
|  |  | **Maximum** | **Minimum** | **Maximum** | **Minimum** |
| **AMR surveillance network** | Government hospital (n=5) | 17,896 | 1,920 | 17,896 | 1,920 |
|  | Private hospital (n=3) | 41,014 | 12,511 | 41,014 | 10,336 |
|  | Public-private (n=3) | 118,667 | 1,262 | 91,844 | 1,166 |
| **Outside network** | Government hospital (n=5) | 56,680 | 1,084 | 56,680 | 1,084 |
|  | Private hospital (n=10) | 50,544 | 14,682 | 37,432 | 12,707 |
|  | Clinic/laboratory (n=2) | 9,657 | 5,048 | 9,657 | 5,048 |

**Supplemental Table 3**: Data completeness for priority variables

|  |  | **Government hospital (%)** | **Private hospital (%)** | **Clinics/laboratory (%)** | **Public/private hospitals (%)** |
| --- | --- | --- | --- | --- | --- |
| **CAPTURA priority variable** | AMR surveillance network | 45.5 | 63.6 | 0 | 54.6 |
|  | Outside network | 45.5 | 45.5 | 54.6 | 0 |
| **GLASS priority variable** | AMR surveillance network | 70 | 70 | 0 | 70 |
|  | Outside network | 70 | 70 | 80 | 0 |
